# DIF Analysis with Unknown Groups and Anchor Items

**DOI:** 10.1007/s11336-024-09948-7

**Published:** 2024-02-21

**Authors:** Gabriel Wallin, Yunxiao Chen, Irini Moustaki

**Affiliations:** 1 Department of Mathematics and Statistics, Lancaster University, Umeå, Sweden; 2 Department of Statistics, London School of Economics and Political Science, Columbia House, Room 5.16 Houghton Street, London, WC2A 2AE UK

**Keywords:** differential item functioning, measurement invariance, latent DIF, latent class analysis, lasso

## Abstract

Ensuring fairness in instruments like survey questionnaires or educational tests is crucial. One way to address this is by a Differential Item Functioning (DIF) analysis, which examines if different subgroups respond differently to a particular item, controlling for their overall latent construct level. DIF analysis is typically conducted to assess measurement invariance at the item level. Traditional DIF analysis methods require knowing the comparison groups (reference and focal groups) and anchor items (a subset of DIF-free items). Such prior knowledge may not always be available, and psychometric methods have been proposed for DIF analysis when one piece of information is unknown. More specifically, when the comparison groups are unknown while anchor items are known, latent DIF analysis methods have been proposed that estimate the unknown groups by latent classes. When anchor items are unknown while comparison groups are known, methods have also been proposed, typically under a sparsity assumption – the number of DIF items is not too large. However, DIF analysis when both pieces of information are unknown has not received much attention. This paper proposes a general statistical framework under this setting. In the proposed framework, we model the unknown groups by latent classes and introduce item-specific DIF parameters to capture the DIF effects. Assuming the number of DIF items is relatively small, an $L_1$-regularised estimator is proposed to simultaneously identify the latent classes and the DIF items. A computationally efficient Expectation-Maximisation (EM) algorithm is developed to solve the non-smooth optimisation problem for the regularised estimator. The performance of the proposed method is evaluated by simulation studies and an application to item response data from a real-world educational test.

**Supplementary Information:**

The online version contains supplementary material available at 10.1007/s11336-024-09948-7.

Psychometric models to analyse data from instruments such as survey questionnaires and educational tests rely on an equivalence assumption on the item parameters across groups of respondents. That is, conditioning on the latent construct measured by the instrument, a respondent’s response to each item is independent of their group membership. This assumption is known as measurement invariance. If violated, the psychometric property of the item(s) is not constant across groups, which can cause measurement bias (Millsap, 2012). The measurement invariance assumption is typically investigated through differential item functioning (DIF) analysis, a class of statistical methods that compares respondent groups at the item level and detects non-invariant, i.e., DIF, items.

Traditional DIF detection methods assume that both the comparison groups and a set of non-DIF items, commonly referred to as the anchor set, are known a priori. The anchor items are used to identify the latent construct the instrument measures, and a DIF detection method compares the performances of the comparison groups, controlling for their performance on the anchor items as a proxy of the latent construct level. Depending on their specific assumptions, these DIF detection methods can be divided into Item-Response-Theory-based (IRT-based) methods (e.g., Kim et al., 1995; Lord, 1977; Lord, 1980; Steenkamp & Baumgartner, 1988; Tay et al., 2012; Thissen et al., 1988; Thissen & Steinberg, 1998; Wainer, 2016; Woods et al., 2013) and non-IRT-based methods (e.g., Cao et al., 2017; Dorans & Kulick, 1986; Drabinová & Martinková, 2017; Holland & Thayer, 1986; Holland & Wainer, 1993; Shealy & Stout, 1993; Swaminathan & Rogers, 1990; Tay et al., 2015; Woods et al., 2013; Zwick et al., 2000); see Millsap (2012) for a review of traditional DIF analysis methods. Generally speaking, IRT-based methods tend to provide a clearer definition of DIF effects through a generative probabilistic model at the price of a risk of model misspecification.

Unfortunately, comparison groups and anchor items may not always be available in real-world applications, in which cases the aforementioned traditional methods are not applicable. Even if we have some information about anchor items, the result may be sensitive to the specific anchor items we use, when we only have a small number of such, and there will be a big issue if the anchor items are misspecified. Modern DIF analysis methods have been developed in situations where either the comparison groups or the anchor items are unknown. When anchor items are unknown, the latent construct is not identified, in which case, DIF detection is an ill-posed problem if no additional assumptions are made. A reasonable assumption in this situation is sparsity – the number of DIF items is relatively small, under which the detection of DIF items is turned into a model selection problem. To tackle the model selection problem, item purification methods have been proposed (e.g., Candell & Drasgow, 1988; Clauser et al., 1993; Fidalgo et al., 2000; Kopf et al., 2015a; b; Wang et al., 2009; Wang & Su, 2004; Wang & Yeh, 2003), where stepwise model selection methods are used to detect DIF items. More recently, Lasso-type regularised estimation methods have been proposed to solve the model selection problem (Magis et al., 2015; Tutz and Schauberger, 2015; Belzak and Bauer, 2020; Bauer et al., 2020; Schauberger and Mair, 2020). In these methods, the DIF effects are represented by item-specific parameters under an IRT model, where a zero coefficient encodes no DIF effect for an item, and Lasso-type penalties are imposed on the DIF parameters to obtain a sparse solution, i.e., many items are DIF-free. A drawback of regularised estimation methods is that, due to the bias brought by Lasso regularisation, they do not provide valid *p*-values for testing whether each item is DIF-free. Recently, Chen et al. (2023) considered a limiting case of a regularised estimator and showed that the estimator can simultaneously identify the latent construct and yield valid statistical inferences on the individual DIF effects. An alternative direction of DIF analysis without anchor items is based on the idea of differential item pair functioning. Under the Rasch model, Bechger and Maris (2015) showed that although a Rasch model with group-specific difficulty parameters is not identifiable, the relative difficulties of item pairs are identifiable and can be used for detecting DIF items. Based on this idea, Yuan et al. (2021) introduced visualisation methods for DIF detection. We lastly point out that there is related literature on $L_1$ regularisation for general mixture models such as Gaussian mixture models (e.g., Bhattacharya & McNicholas, 2014; Bouveyron & Brunet-Saumard, 2014; Luo et al., 2008), which also consider model-based clustering but are based on continuous instead of categorical data. However, it is worth noting that these works all consider a high-dimensional data setting, and the regularisation is used for dimension reduction. The current paper focuses on a relatively low-dimensional setting, and a regularised estimator is proposed for the purpose of model selection.

The comparison groups may sometimes be unavailable, and DIF analysis in this situation is typically referred to as latent DIF analysis (Cho et al., 2016; De Boeck et al., 2011). As suggested in De Boeck et al. (2011), latent DIF analysis is needed when we do not know the crucial groups for comparison, we cannot observe the groups of interest, or there are validity concerns regarding the true group membership of the respondents. For example, for self-reported health and mental health instruments (Teresi and Reeve, 2016; Reeve and Teresi, 2016; Teresi et al., 2021), many covariates are collected, such as age, gender, ethnicity, and other background variables, but the crucial groups for DIF analysis are typically unclear. For another example, when analysing data from an educational test in which a subset of test takers have preknowledge on some leaked items (Cizek and Wollack, 2017), the two comparison groups of interest – the ones with and without item preknowledge – are not directly observable. Moreover, the observed group membership may sometimes poorly indicate the “true” group membership that causes the DIF pattern in the item response data (e.g., Bennink et al., 2014; Cho & Cohen, 2010; Finch & Hernández Finch, 2013; Von Davier et al., 2011). Most existing latent DIF analysis methods assume that an anchor set is known and use a mixture IRT model – a model that combines IRT and latent class analysis– to identify the unknown groups and detect the DIF items simultaneously (Cho and Cohen, 2010; Cohen and Bolt, 2005; De Boeck et al., 2011); see Cho et al. (2016) for a review.

In practice, both the comparison groups and the anchor set may be unknown. For example, besides the aforementioned challenges of identifying the crucial comparison groups, the DIF analysis of self-reported health and mental health instruments also faces the challenge of identifying anchor items (Teresi and Reeve, 2016; Reeve and Teresi, 2016). In the item preknowledge example above, not only the comparison groups are unobserved, but also prior knowledge about non-leaked items is likely unavailable, and thus, correctly specifying an anchor set is a challenge (O’Leary et al., 2016). Almost no general methods are available for latent DIF analysis when the anchor set is unavailable. Two notable exceptions are Chen et al. (2022) and Robitzsch (2022). In Chen et al. (2022), a Bayesian hierarchical model for latent DIF analysis is proposed and applied for the simultaneous detection of item leakage and preknowledge in educational tests. In this model, latent classes are imposed among the test takers to model the comparison groups, and also among the items to model the DIF and non-DIF item sets. In addition, both the person- and item-specific parameters are treated as random variables and inferred via a fully Bayesian approach. However, the inference of this model relies on a Markov chain Monte Carlo algorithm, which suffers from slow mixing. Moreover, as most traditional DIF analysis methods adopt a frequentist setting, it is of interest to develop a frequentist approach to latent DIF analysis when the anchor set is unknown. Robitzsch (2022) proposed a latent DIF procedure based on a regularised estimator under a mixture Rasch model. In this work, a nonconvex penalty called the Smoothly Clipped Absolute Deviation (SCAD) penalty (Fan and Li, 2001) other than the $L_1$ penalty is investigated. The methodology proposed in the current paper is similar in spirit to that of Robitzsch (2022) but developed independently. The proposed framework focuses on the two-parameter logistic (2-PL) model (Birnbaum, 1968) with an $L_1$ penalty and further provides a scope to generalise to other item response theory models.

This paper proposes a frequentist framework for DIF analysis when both the comparison groups and the anchor set are unknown. The proposed framework combines the ideas of mixture IRT modeling for latent DIF analysis and regularised estimation for manifest DIF analysis with unknown anchor items. More specifically, the unknown groups are modelled by latent classes, and the DIF effects are characterised by item-specific DIF parameters. An $L_1$-regularised marginal maximum likelihood estimator is proposed, assuming that the number of DIF items is relatively small. This estimator penalises the DIF parameters by a Lasso regularisation term so that the DIF items can be selected by the non-zero pattern of the estimated DIF parameters. Computing the $L_1$-regularised estimator involves solving a non-smooth optimisation problem. We propose a computationally efficient Expectation-Maximisation (EM) algorithm (Dempster et al., 1977; Bock and Aitkin, 1981), where the non-smoothness of the objective function is handled by a proximal gradient method (Parikh and Boyd, 2014). We evaluate the proposed method through simulation studies and an application to item response data from a real-world educational test. For the real-world application, we consider data from a midwestern university in the United States. This data set has been studied in Bolt et al. (2002), where end-of-test items are believed to cause DIF due to insufficient time. Both the comparison groups, i.e. the speeded and non-speeded respondents, and the anchor items are unknown. In Bolt et al. (2002), the DIF items and comparison groups are detected by borrowing information from an additional test form which is carefully designed so that the potential speededness-DIF items in the original form are administered at earlier locations, and thus, are unlikely to suffer from speededness-DIF. Thanks to the proposed procedure, we are able to identify the unknown DIF items and comparison groups without utilising information from the additional test form, and our findings are consistent with those of Bolt et al. (2002).

The rest of the paper is organised as follows. In Sect. 1, we propose a modelling framework for latent DIF analysis with unknown groups and anchor items and a regularised estimator that simultaneously identifies the unknown groups and detects the DIF items. In Sect. 2, we propose a computationally efficient EM algorithm. The proposed method is evaluated by simulation studies in Sect. 3 and further applied to data from a real-world educational test in Sect. 4. We conclude with discussions in Sect. 5. Details about the computational algorithm are given in the Appendix.

## Proposed Framework

### Measurement Model

Consider *N* respondents answering *J* binary items. Let $Y_{ij} \in \{0, 1\}$ for $i = 1, \ldots , N$ and $j = 1, \ldots , J$ be a binary random variable recording individual *i*’s response to item *j*. The response vector of individual *i* is denoted by ${\textbf{Y}}_i = (Y_{i1}, \ldots , Y_{iJ})^\top$. We assume that the items measure a unidimensional construct, which is modelled by a latent variable $\theta _i$. We further assume that the respondents are random samples from $K+1$ unobserved groups, where the group membership is denoted by the latent variable $\xi _i \in \{0, 1,..., K\}$. Given the latent trait $\theta _i$ and the latent class $\xi _i$, consider the two-parameter item response model with a logit link (2-PL) (measurement model) (Birnbaum, 1968)

1 $$\begin{aligned} \text {logit} P(Y_{ij} = 1|\theta _i, \xi _i ) = a_j \theta _i + d_j+ \delta _{j\xi _i}, \end{aligned}$$

where $a_j$ and $d_j$ are known as the discrimination and easiness parameters respectively and $\delta _{j \xi _i}$ is referred to as the DIF-effect parameter, as it quantifies the DIF effect of latent class *k* on item *j*.

We treat $\xi _i = 0$ as the baseline group, also known as the reference group, and set $\delta _{j0} = 0$ for all $j = 1, \ldots , J$. In that case, $a_j \theta _i + d_j$ denotes the item response function for the reference group. When $a_j$ is common across all items, the baseline model becomes the Rasch model (Rasch, 1960). We focus on the 2-PL model here, but the proposed method easily adapts to other baseline IRT models.

The parameter $\delta _{jk}$ characterises how respondents in group *k* differ from those in the reference group in terms of the item response behaviour on item *j*. For the reference group, the DIF parameter remains zero for all items, serving as a reference point. For the remaining latent classes, the DIF parameter can be non-zero for certain items. Crucially, the magnitude of this parameter is allowed to differ across these latent classes. This flexibility accounts for varying degrees of DIF effects across different latent groups, when comparing with the reference group. The DIF effect parameter can also be expressed in terms of log-odds. Specifically, under the 2-PL model,

$$\begin{aligned} \delta _{jk} = \log \left( \frac{P(Y_{ij} = 1|\theta _i = \theta , \xi _i = k)/(1-P(Y_{ij} = 1|\theta _i = \theta , \xi _i = k))}{P(Y_{ij} = 1|\theta _i = \theta , \xi _i = 0)/(1-P(Y_{ij} = 1|\theta _i = \theta , \xi _i = 0))}\right) , \end{aligned}$$

i.e., $\delta _{jk}$ is the log-odds-ratio when comparing two respondents from group *k* and the reference group given that they have the same latent construct level.

### Structural Model

The structural model specifies the joint distribution of the latent variables $(\theta _i, \xi _i)$. We assume that the latent classes follow a categorical distribution,

$$\begin{aligned} \xi _i \sim \text {Categorical}(\{0, 1, \ldots , K\}, (\nu _0, \nu _1, \ldots , \nu _K)), \end{aligned}$$

where $P(\xi _i = k) = \nu _k$. There are consequently $K+1$ latent classes with class probabilities $\varvec{\nu }= (\nu _0, \nu _1, \ldots , \nu _K)^\top$ such that $\nu _k \ge 0$ and $\sum _{k=0}^{K} \nu _k = 1$. We further assume that conditional on $\xi _i$, the latent ability $\theta _i$ follows a normal distribution with class-specific mean and variance, i.e.,

$$\begin{aligned} \theta _i | \xi _i = k \sim {\mathcal {N}}(\mu _k, \sigma _k^2). \end{aligned}$$

To ensure model identification, we fix the mean and variance of the reference group, i.e. $\mu _0 = 0$ and $\sigma _0^2 = 1$.

**Fig. 1:**
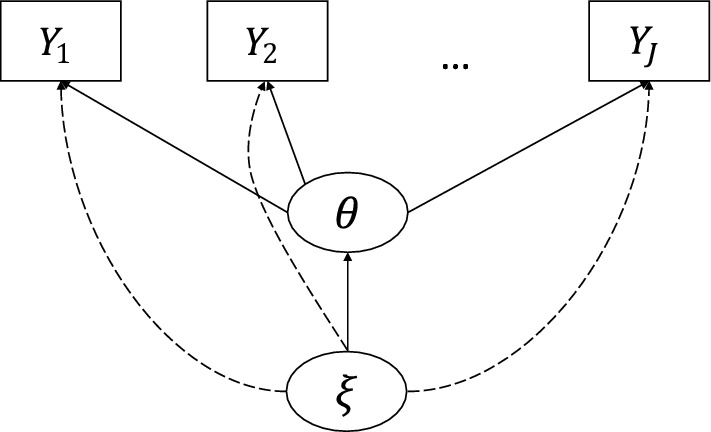
Path diagram of the proposed model, where the dashed lines indicate the DIF effects.

The path diagram of this model is given in Fig. 1. We note that the model coincides with a MIMIC model (Jöreskog and Goldberger, 1975) for manifest DIF analysis (Muthen and Lehman, 1985; Muthén, 1989; Woods, 2009; Woods and Grimm, 2011) when conditioning on the latent class $\xi _i$ (i.e., viewing $\xi _i$ as observed). However, since $\xi _i$ is unobserved, the statistical inference of the proposed model differs substantially from that of the MIMIC model. More specifically, the inference of the proposed model will be based on the marginal likelihood function where both latent variables $\xi _i$ and $\theta _i$ are marginalised out. When the baseline IRT model is the 2-PL model, the marginal likelihood function takes the form

2 $$\begin{aligned} \begin{aligned} L(\Delta )&= \prod _{i=1}^N \sum _{k=0}^K \nu _k \int \left( \prod _{j=1}^J \left( \exp ((a_j\theta + d_j + \delta _{jk})Y_{ij})/(1+\exp (a_j\theta + d_j + \delta _{jk}))\right) \right) \\&\quad \phi (\theta \vert \mu _k, \sigma ^2_k) d\theta , \end{aligned} \end{aligned}$$

where $\phi (\theta \vert \mu _k, \sigma ^2_k)$ denotes the density function of a normal distribution with mean $\mu _k$ and variance $\sigma _k^2$, and we use vector $\Delta$ to denote all the unknown parameters, including the item parameters $a_j$ and $d_j$, $\delta _{jk}$, $\nu _k$, $\mu _k$ and $\sigma _k^2$, for $j = 1,..., J$ and $k = 0, 1,..., K$.

### Model Identifiability

The current model suffers from two sources of unidentifiability. The first source of unidentifiability comes from not knowing the anchoring items, which occurs even if we condition on the latent class $\xi _i$, i.e., when the model becomes a MIMIC model for manifest DIF analysis. That is, for any constants $c_1,..., c_K$, if we simultaneously replace $\mu _k$ by $\mu _k + c_k$ and replace $\delta _{jk}$ by $\delta _{jk} - a_j \mu _k$ for all $j = 1,..., J$ and $k=1,..., K$, the likelihood function value $L(\Delta )$ remains the same. This source of unidentifiability can be avoided when one or more anchor items are known a priori. Suppose that item *j* is known to be DIF-free. Under the proposed model framework, it implies the constraints $\delta _{jk} = 0$ for all *k*. Consequently, the aforementioned transformation can no longer apply, as otherwise, the zero constraints for the anchor items will be violated. As discussed in Sect. 1.4, this source of unidentifiability can be handled by a regularised estimation approach under a sparsity assumption that many DIF parameters $\delta _{jk}$ are zero.

The second source of unidentifiability is the label-switching phenomenon of latent class models (Redner and Walker, 1984), as a result of the exchangeability of the latent classes. Under the current model, the baseline class is uniquely identified through the constraints $\delta _{j0} =0$, $\mu _0 = 0$ and $\sigma _0^2 = 1$. However, the remaining latent classes are exchangeable, and the likelihood function value remains the same when switching their labels. While label switching often causes trouble when inferring a latent class model with Bayesian Markov chain Monte Carlo (MCMC) algorithms (Stephens, 2000), it is not a problem for the estimator to be discussed in Sect. 1.4. Our estimator is proposed under the frequentist setting and computed by an EM algorithm. When the EM algorithm converges, it will reach one of the equivalent solutions in the sense of label switching.

### Sparsity, Model Selection and Estimation

As explained above, the latent trait cannot be identified without anchor items. In that case, additional assumptions are needed to solve the DIF analysis problem. Specifically, we adopt the sparsity assumption, i.e., many DIF parameters $\delta _{jk}$ are zero. This is a common assumption in the manifest DIF literature, see for example (Magis et al., 2015; Tutz and Schauberger, 2015; Belzak and Bauer, 2020; Bauer et al., 2020; Schauberger and Mair, 2020). In many applications, for example, the detection of aberrant behaviour or parameter drift in educational testing, the number of DIF items is low, suggesting that this assumption is meaningful.

Under the above sparsity assumption, we propose an $L_1$ regularised estimator to simultaneously estimate the unknown model parameters and learn the sparsity pattern of the DIF-effect parameters. This estimator takes the form

3 $$\begin{aligned} {\tilde{\Delta }}^{(\lambda )} = \mathop {\text {arg min}}\limits _{\Delta } -\log L(\Delta ) + \lambda \sum _{j=1}^J\sum _{k=1}^K |\delta _{jk}|, \text{ s.t. } \nu _k \ge 0, k=0, 1,..., K, \text{ and } \sum _{k=0}^K \nu _k =1,\nonumber \\ \end{aligned}$$

where $L(\Delta )$ is the marginal likelihood function defined in (2), and $\lambda > 0$ is a tuning parameter. The computation of this estimator will be discussed in Sect. 2. Similar to Lasso regression (Tibshirani, 1996), the $L_1$ regularisation term $\lambda \sum _{j=1}^J \sum _{k=1}^K|\delta _{jk}|$ in (3) tends to shrink some of the DIF-effect parameters to be exactly zero. In the most extreme case where $\lambda$ goes to infinity, all the DIF-effect parameters will shrink to zero. Under suitable regularity conditions and when $\lambda$ is chosen properly (i.e., $\lambda$ goes to infinity at a suitable speed), the $L_1$ regularised estimator yields both estimation and selection consistency (Zhao and Yu, 2006; van de Geer, 2008). In that case, the latent trait is consistently identified, and the consistently selected sparse patterns of the estimated DIF-effect parameters can be used to classify items as DIF and non-DIF items.

We select the tuning parameter $\lambda$ based on the Bayesian Information Criterion (BIC; Schwarz, 1978) using a grid search approach. Specifically, we consider a pre-specified set of grid points for $\lambda$, denoted by

$\lambda _1$,... $\lambda _{M}$. For each value of $\lambda _m$, we solve the optimisation problem (3) and obtain ${\tilde{\Delta }}^{(\lambda _m)}$. To compute the BIC value for the model encoded by ${\tilde{\Delta }}^{(\lambda _m)}$, we compute a constrained maximum likelihood estimator, fixing $\delta _{jk}$ to zero if ${\tilde{\delta }}_{jk}^{(\lambda _{m})} = 0$. That is,

4 $$\begin{aligned} \begin{aligned} {\hat{\Delta }}^{(\lambda _m)}&= \mathop {\text {arg min}}\limits _{\Delta } -\log L(\Delta ), \\&\quad \text{ s.t. } \nu _k \ge 0, k =0,..., K, ~ \sum _{k=0}^K \nu _k =1, \\&\quad \delta _{jk} = 0 \text{ if } {\tilde{\delta }}_{jk}^{(\lambda _{m})} = 0, j = 1,..., J, k=1,..., K. \end{aligned} \end{aligned}$$

The BIC corresponding to $\lambda _m$ is calculated as

5 $$\begin{aligned} \text{ BIC}_{\lambda _m} = -2\log L({\hat{\Delta }}^{(\lambda _m)}) + \log (N)\text{ Card }({\hat{\Delta }}^{(\lambda _m)}), \end{aligned}$$

where $\text{ Card }({\hat{\Delta }}^{(\lambda _m)})$ denotes the number of free parameters in ${\hat{\Delta }}^{(\lambda _m)}$ that equals to the total number of free parameters in $\Delta$ minus the corresponding number of zero constraints. The tuning parameter is then selected as

$$\begin{aligned} {{\hat{\lambda }}} = \mathop {\text {arg min}}\limits _{\lambda _m, m=1,..., M} \text{ BIC}_{\lambda _m}. \end{aligned}$$

Thanks to the asymptotic properties of the BIC (Shao, 1997), the true model will be consistently selected if it can be found by one of the tuning parameters.

We use the constrained maximum likelihood estimator ${\hat{\Delta }}^{({{\hat{\lambda }}})}$ as the final estimator of the selected model and declare an item *j* to be a DIF item if $\Vert ({{\hat{\delta }}}_{j1}^{({{\hat{\lambda }}})},..., {{\hat{\delta }}}_{jK}^{({{\hat{\lambda }}})})^\top \Vert \ne 0$. We summarise this procedure in Algorithm 1 below.

**Algorithm 1:**
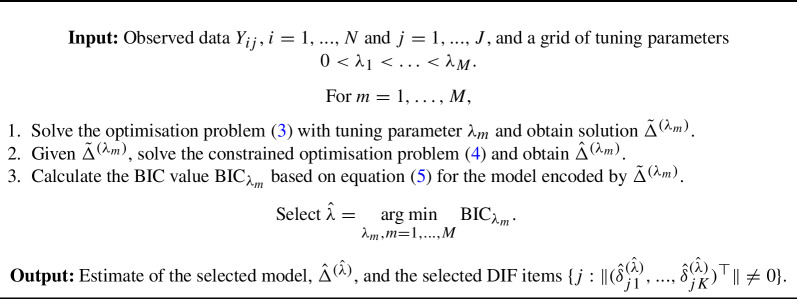
Regularised estimation and model selection.

### Other Inference Problems

The latent class membership can be inferred by an empirical Bayes procedure, i.e., by the maximum a posteriori (MAP) estimate under the estimated model. For the MAP estimator, the goal is to find the most probable latent class *k* for each respondent *i*, given their observed responses ${\varvec{y}}_i$. This is done by maximizing the posterior probability of $\xi _i$ being equal to *k*, conditioned on the observed responses ${\varvec{y}}_i$:

$$\begin{aligned} {\hat{\xi }}_{\text {MAP}, i} = \mathop {\text {arg max}}\limits _{k \in \{0, 1,..., K\}} {\hat{P}}(\hat{\xi } = k|{\textbf{y}}_i) \end{aligned}$$

We get these posterior probabilities through

6 $$\begin{aligned} {\hat{P}}(\xi _i = k | {\textbf{y}}_i) = \frac{{\hat{P}}({\textbf{y}}_i | \xi _i = k, \theta _i) \cdot {\hat{P}}(\xi _i = k)}{{\hat{P}}({\textbf{y}}_i)} \end{aligned}$$

based on the estimated model parameters.

Lastly, the number of latent classes, i.e., the choice of *K*, can be determined using the BIC. That is, we solve the optimisation problem in (3) for different values of *K*, yielding ${\hat{\Delta }}^{(K)}$. We thereafter compute the BIC as

7 $$\begin{aligned} \text{ BIC }(K) = -2\log L({\hat{\Delta }}^{(K)}) + \log (N)\text{ Card }({\hat{\Delta }}^{(K)}), \end{aligned}$$

and select the *K* that yields the smallest BIC:

8 $$\begin{aligned} K^* = \mathop {\text {arg min}}\limits _K \text{ BIC }(K) \end{aligned}$$

### Extensions

The proposed model is possible to extend in several ways to accommodate different data types and more than one factor. To make this clear, our model can be expressed as

9 $$\begin{aligned} P(Y_{ij} = 1 | \theta _i, \xi _i) = f\bigg (g(\theta _i, \beta _j) + \delta _{j \xi _i} \bigg ) \end{aligned}$$

The function *g* determines the parametrisation of the person and item parameters, denoted by $\theta$ and $\beta$ respectively. If for example the Rasch model (Rasch, 1960) is adopted, $\beta _j = (a, d_j)$ where $a_j=a$ for all $j=1, \ldots , J$,

$$\begin{aligned} g(\theta _i, \beta _j) = a\theta _i + d_j, \end{aligned}$$

and $$f(x) = \exp (x)/(1+\exp (x))$$.

It is also possible to consider link functions other than the logistic function considered in this paper, such as the probit link:

$$\begin{aligned} f(x) = \int _{- \infty }^x \phi (z) dz. \end{aligned}$$

DIF analysis using multidimensional IRT models with unknown anchor items has recently been considered in Wang et al. (2023). As in the unidimensional case, no method can handle situations where both the groups and the anchor items are missing. Our proposed framework can however be extended to handle such situations. Consider the extension of (9) where each respondent, in addition to the latent class membership $\xi _i$, is represented by an *L*-dimensional latent vector $\varvec{\theta }_i = (\theta _{i1}, \ldots , \theta _{iL})^\top$. Each item is represented by an intercept parameter $d_j$ and *L* loading parameters ${\varvec{a}}_j = (a_{j1}, \ldots , a_{jL})^\top$. This extension of the model can be expressed as a multidimensional 2-PL model with an added DIF component, i.e.,

$$\begin{aligned} P(Y_{ij} = 1 | \varvec{\theta }_i, \xi _i) = f\bigg (d_j + {\varvec{a}}_j^\top \varvec{\theta }_i + \delta _{j \xi _i} \bigg ). \end{aligned}$$

The proposed modeling framework can also be extended to accommodate ordinal data. Denoting the response categories of $Y_{ij}$ by $c = 1, \ldots , C$, such model can, using the logistic link, be expressed as

$$\begin{aligned} \text {logit} P(Y_{ij} \le c) = d_{jc} - a_j \theta _i + \delta _{j \xi _i} \end{aligned}$$

The model without the DIF parameter is known as the proportional odds model (Samejima, 1969). Note the negative sign in front of the slope parameter so that if $a_j$ is positive, increasing $\theta _i$ will increase the probability of higher-numbered levels of $Y_{ij}$.

Lastly, we mention the possibility of extending the model to accommodate for DIF effects in the discrimination parameter, known as non-uniform DIF. To consider such a case, we introduce a similar DIF-effect parameter for $a_j$, just as we have for $d_j$. Let’s denote the DIF effect on the discrimination parameter $\alpha _{j \xi _i}$. Given that $\xi _i=0$ is treated as the reference group, we set $\alpha _{j0}=0$ for all $j=1,\ldots , J$. In this case, the modified 2-PL model with a logit link which accounts for DIF in both $a_j$ and $d_j$ can be written as:

10 $$\begin{aligned} \text {logit} P(Y_{ij} = 1|\theta _i, \xi _i ) = (a_j + \alpha _{j\xi _i}) \theta _i + d_j + \delta _{j\xi _i}. \end{aligned}$$

To include DIF in the $L_1$ regularised estimator, the penalty term needs to be modified to penalize both $\alpha _{jk}$ and $\delta _{jk}$ terms. The modified estimator is given by

11 $$\begin{aligned} {\tilde{\Delta }}^{(\lambda )}= & {} \mathop {\text {arg min}}\limits _{\Delta } -\log L(\Delta ) + \lambda \left( \sum _{j=1}^J\sum _{k=1}^K |\delta _{jk}| + \sum _{j=1}^J\sum _{k=1}^K |\alpha _{jk}| \right) ,\nonumber \\{} & {} \text{ s.t. } \,\, \nu _k \ge 0, k=0, 1,..., K, \text{ and } \sum _{k=0}^K \nu _k =1, \end{aligned}$$

## Computation

The computation of the optimisation problems (3) and (4) is carried out using the EM algorithm (Dempster et al., 1977; Bock and Aitkin, 1981). An EM algorithm is an iterative algorithm, alternating between an Expectation (E) step and a Maximisation (M) step. Optimisation problem (4) involves maximising the marginal likelihood function of a regular latent variable model, and thus, can be solved by a standard EM algorithm. Thus, its details are skipped here. However, the optimisation problem (3) involves a non-smooth $L_1$ term. Consequently, the M step of the algorithm cannot be carried out using a gradient-based numerical solver, such as a Newton-Raphson algorithm. We develop an efficient proximal-gradient-based EM algorithm that uses a proximal gradient update (Parikh and Boyd, 2014) to carry out the non-smooth optimisation problem in the M-step. In what follows, we elaborate on this algorithm using the 2-PL model as the baseline IRT model, while pointing out that the algorithm easily extends to other baseline IRT models.

Suppose that *t* iterations of the algorithm have been run and let $\Delta ^{(t)}$ be the current parameter value. In the E-step of the *t*th iteration, we construct a local approximation of the negative objective function at $\Delta ^{(t)}$ in the form of

12 $$\begin{aligned} Q(\Delta | \Delta ^{(t)})= & {} \sum _{i=1}^N {\mathbb {E}}\left[ \log \left( \nu _{\xi _i}\prod _{j=1}^J \left( \frac{\exp ((a_j\theta _i + d_j + \delta _{j\xi _i})Y_{ij})}{1+\exp (a_j\theta _i + d_j + \delta _{j\xi _i})}\right) \phi (\theta _i \vert \mu _{\xi _i}, \sigma ^2_{\xi _i}) \big \vert {\textbf{Y}}_i, \Delta ^{(t)}\right) \right] \nonumber \\{} & {} - \lambda \sum _{j=1}^J\sum _{k=1}^K |\delta _{jk}|. \end{aligned}$$

We note that the expectation in (12) is with respect to the conditional distribution of the latent variables $(\theta _i, \xi _i)$ given ${\textbf{Y}}_i$, evaluated at the current parameters $\Delta ^{(t)}$.

In the M-step, we find $\Delta ^{(t+1)}$ such that

$$\begin{aligned} Q(\Delta ^{(t+1)} | \Delta ^{(t)}) > Q(\Delta | \Delta ^{(t)}), \end{aligned}$$

or equivalently,

$$\begin{aligned} -Q(\Delta ^{(t+1)} | \Delta ^{(t)}) < -Q(\Delta | \Delta ^{(t)}). \end{aligned}$$

By Jensen’s inequality, it consequently guarantees that the objective function of (3) decreases, i.e.,

$$\begin{aligned} -\log L(\Delta ^{(t+1)}) + \lambda \sum _{j=1}^J\sum _{k=1}^K |\delta _{jk}^{(t+1)}| < -\log L(\Delta ^{(t)}) + \lambda \sum _{j=1}^J\sum _{k=1}^K |\delta _{jk}^{(t)}|. \end{aligned}$$

More specifically, we write $\Delta = (\Delta _1^\top , \Delta _2^\top )^\top ,$ where $\Delta _1 = (\nu _0,..., \nu _K)^\top$ and $\Delta _2$ contains the rest of the parameters. We notice that $-Q(\Delta | \Delta ^{(t)})$ in (12) can be decomposed as the sum of a smooth function

$D_t(\Delta _1) = -\sum _{i=1}^N {\mathbb {E}}\left[ \log \left( \nu _{\xi _i} \big \vert {\textbf{Y}}_i, \Delta ^{(t)}\right) \right] ,$ a smooth function $F_t(\Delta _2)$, defined as

$$\begin{aligned} F_t(\Delta _2) = -\sum _{i=1}^N {\mathbb {E}}\left[ \log \left( \prod _{j=1}^J \left( \frac{\exp ((a_j\theta _i + d_j + \delta _{j\xi _i})Y_{ij})}{1+\exp (a_j\theta _i + d_j + \delta _{j\xi _i})}\right) \phi (\theta _i \vert \mu _{\xi _i}, \sigma ^2_{\xi _i}) \big \vert {\textbf{Y}}_i, \Delta ^{(t)}_2\right) \right] \end{aligned}$$

and a non-smooth function $G(\Delta _2) = \lambda \sum _{j=1}^J\sum _{k=1}^K |\delta _{jk}|.$ We note that $\Delta _1^{(t+1)}$ can be obtained by solving the following constrained optimisation problem

$$\begin{aligned} \Delta _1^{(t+1)} = \mathop {\text {arg min}}\limits _{\Delta _1} D_t(\Delta _1), \text{ s.t. } \nu _k \ge 0, k=0, 1,..., K, \text{ and } \sum _{k=0}^K \nu _k =1. \end{aligned}$$

Using the method of Lagrangian multiplier, this optimisation problem has a closed-form solution; see the Appendix for the details.

We then find $\Delta _2^{(t+1)}$ such that $F_t(\Delta _2^{(t+1)}) + G(\Delta _2^{(t+1)}) < F_t(\Delta _2^{(t)}) + G(\Delta _2^{(t)})$. Consider the optimisation problem $\min F_t(\Delta _2) + G(\Delta _2)$. Due to the non-smoothness of *G*, this objective function is not differentiable everywhere. Consequently, gradient-based methods are not applicable. We find $\Delta _2^{(t+1)}$ by using a proximal gradient method (Parikh and Boyd, 2014). Denote the dimension of $\Delta _2$ by *d*, where *d* equals the number of free parameters which is determined by the choice of baseline model ($2J + 2K$ for the 2-PL model). We define the proximal operator of *G* at $\Delta _2$ as

$$\begin{aligned} \text{ Prox}_G(\Delta _2) = \mathop {\text {arg min}}\limits _{{{\tilde{\Delta }}}_2 \in {\mathbb {R}}^d} ~G(\Delta _2) + \frac{1}{2} \Vert {{\tilde{\Delta }}}_2 - \Delta _2\Vert ^2. \end{aligned}$$

We update $\Delta _2$ by

13 $$\begin{aligned} \Delta _2^{(t+1)} = \text{ Prox}_{\alpha G}(\Delta _2^{(t)} - \alpha \nabla F_t(\Delta _2^{(t)})), \end{aligned}$$

where $\nabla F_t(\Delta _2^{(t)})$ denotes the gradient of $F_t$ at $\Delta _2^{(t)}$, and $\alpha$ is a step size. According to Parikh and Boyd (2014, sect.4.2), for a sufficiently small step size $\alpha$, it is guaranteed that $F_t(\Delta _2^{(t+1)}) + G(\Delta _2^{(t+1)}) < F_t(\Delta _2^{(t)}) + G(\Delta _2^{(t)})$, unless $\Delta _2^{(t)}$ is already a stationary point. Thus, we select $\alpha$ by a line search procedure, whose details are provided in the Appendix. We note that this proximal operator has a closed-form solution. Specifically, each DIF-effect parameter $\delta _{jk}$ is updated by solving

$$\begin{aligned} \delta _{jk}^{(t+1)} = \mathop {\text {arg min}}\limits _{{{\tilde{\delta }}}_{jk}} -\frac{1}{2}\left( {{\tilde{\delta }}}_{jk} - \left( \delta _{jk}^{(t)} - \alpha \frac{\partial F_t(\Delta _2)}{\partial \delta _{jk}} \big \vert _{\Delta _2 = \Delta _2^{(t)}}\right) \right) ^2 + \alpha \lambda |{{\tilde{\delta }}}_{jk}|, \end{aligned}$$

which has a closed-form solution given by soft-thresholding (Chapter 3, Hastie et al., 2009). The rest of the parameters in $\Delta _2$ do not appear in the non-smooth function $G(\Delta _2)$, and thus, the resulting update of (13) degenerates to a vanilla gradient descent update. For example,

$$\begin{aligned} d_j^{(t+1)} = d_j^{(t)} - \alpha \frac{\partial F_t(\Delta _2)}{\partial d_{j}} \big \vert _{\Delta _2 = \Delta _2^{(t)}}. \end{aligned}$$

Further details of the proximal gradient update can be found in the Appendix. We summarise the main steps of this EM algorithm in Algorithm 2 below.

**Algorithm 2:**
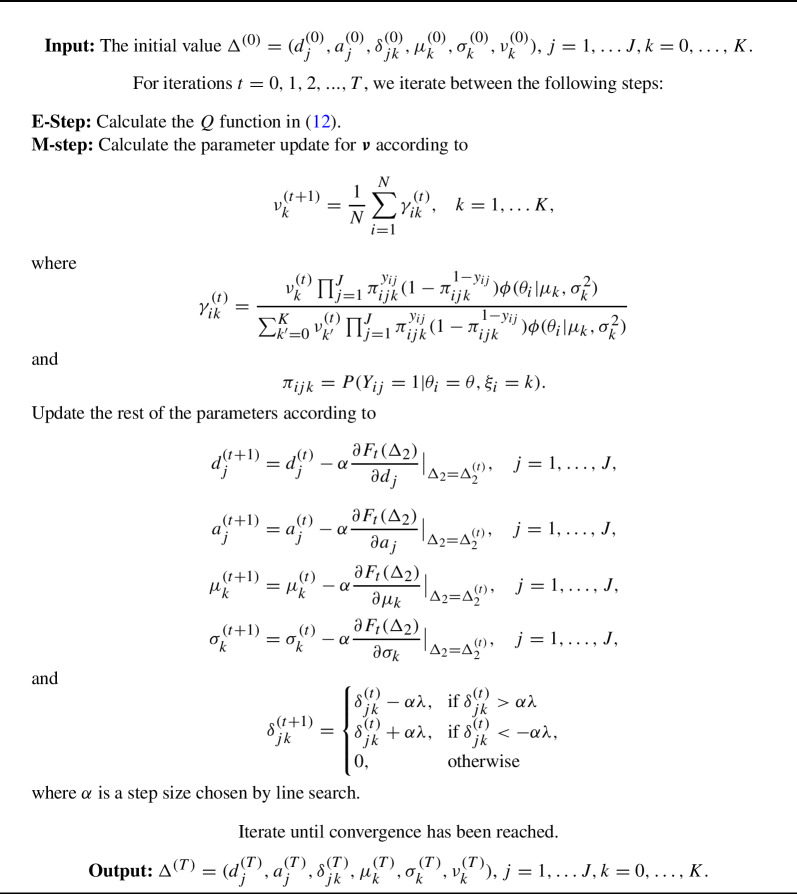
An EM algorithm for solving (3).

*Remark. While differentiable approximations, such as the smoothed Lasso, can allow the use of gradient-based methods, they often come with their own set of challenges. Introducing a smoothing parameter can make the method sensitive to its choice, and in some situations, the approximation might not be close enough to the original problem, especially when the smoothing parameter is not sufficiently small. We opt for an approach based on non-smooth optimisation. We believe that directly tackling the non-smoothness ensures that we do not compromise on the sparsity of the solution, which is critical for our analysis. We have designed our algorithm around the EM algorithm, which inherently possesses certain convergence properties. Specifically, the EM algorithm is guaranteed to increase the log-likelihood in each iteration, and under mild regularity conditions, it converges to at least a local maximum of the log-likelihood. While we acknowledge that there are potential pitfalls in the convergence of non-smooth optimisation algorithms, we have implemented strategies to ensure the stability of our algorithm, such as adaptive step sizes and convergence checks.*

## Simulation Study

In this simulation, we evaluate the performance of the proposed method, treating the number of latent classes *K* as fixed. We consider cases with two and three latent classes, respectively. For each simulation scenario, we run $B = 100$ independent replications.

### Settings

We examine 16 scenarios under the two-group setting and 8 scenarios under the three-group setting, considering $J \in \{25, 50\}$, $N \in \{500, 1000\}$, and $K \in \{1, 2\}$. Note that $K=1$ represents a two-group setting and $K=2$ indicates a three-group setting. In the two-group setting, the class proportions are varied, with the reference group consisting of either 50%, 80%, or 90% of the respondents. In the three-group scenario, 50% of the respondents belong to the reference group, 30% belong to the second latent class, and 20% belong to the third latent class. The number of DIF items is set to 10 for all cases with $J=25$ and 20 for all cases with $J=50$, i.e., the proportion of DIF items remains the same.

The intercept parameters $d_j$ are generated from the $\text {Uniform}(-2, 2)$ distribution and the slope parameters $a_j$ from the $\text {Uniform}(0.5, 1.5)$ distribution. In the two-group setting, we consider three cases for the class proportions, where $\nu = (0.1, 0.9)$, $\nu = (0.2, 0.8)$, and $\nu = (0.5, 0.5)$, respectively. The latent construct $\theta$ within each latent class $\xi$ follows

$$\begin{aligned} \theta _i|\xi = 0 \sim {\mathcal {N}}(0, 1) \,\,\,\, \text {and} \,\,\,\, \theta _i|\xi = 1 \sim {\mathcal {N}}(0.5, 1.5), \end{aligned}$$

and for the three-group case, we additionally let $\theta _i|\xi = 2 \sim {\mathcal {N}}(1, 1.2)$. The DIF effect parameters are generated as $\delta _{jk} \sim \text {Uniform}(0.5, 1.5)$ for the non-zero elements and set to 0 for the remaining items. For the three-group case, the DIF effects for the second and third latent class are generated from a $\text {Uniform}(0.5, 1)$ and $\text {Uniform}(1, 1.5)$ distribution, respectively. In the two-group setting, the DIF items are positioned at the beginning of the scale (items 1-10 when $J=25$ and 1-20 when $J=50$). In the three-group setting, the DIF items are positioned at the end of the scale (items 16-25 when $J=25$ and 31-50 when $J=50$). The true model parameters are given in the supplementary material.

### Evaluation Criteria

**Detection of DIF items.** We check whether the DIF items can be accurately detected by the proposed method. In this analysis, we assume the number of latent classes *K* is known. We calculate the average True Positive Rate (TPR) as

14 $$\begin{aligned} \overline{\text {TPR}} = \frac{1}{B} \sum _{b=1}^B\frac{\sum _{j,k}\mathbb {1}_{ \{{\hat{\delta }}^{(b)}_{jk} \ne 0, \delta _{jk}^{\!{*}} \ne 0 \}} }{\sum _{j,k} \mathbb {1}_{\{\delta _{jk}^{\!{*}} \ne 0\}}}, \end{aligned}$$

where $\{ {\hat{\delta }}^{(b)}_{jk} \}_{J \times K}$ is the estimated DIF effect matrix in the *b*-th replication and $\{\delta _{jk}^{\!{*}}\}_{J\times K}$ denotes the true DIF effect matrix. Similarly, we calculate the average true negative rate ($\overline{\text {TNR}}$), which is the failure rate of identifying zero elements:

15 $$\begin{aligned} \overline{\text {FPR}} = \frac{1}{B} \sum _{b=1}^B \frac{\sum _{j,k}\mathbb {1}_{ \{{\hat{\delta }}^{(b)}_{jk} \ne 0, \delta _{jk}^{\!{*}} = 0 \}} }{\sum _{j,k} \mathbb {1}_{\{\delta _{jk}^{\!{*}} = 0\}}}. \end{aligned}$$

To better evaluate the performance of the proposed method in detecting DIF items, we compare the FPR and TPR with the results under an oracle setting where the group membership $\xi _i$ is known while anchor items are unknown. Under this oracle setting, we solve the following regularised estimation problem as in Bauer et al. (2020):

16 $$\begin{aligned} \min _{\Xi } -\log L^{ora}(\Xi ) + \lambda \sum _{j=1}^J\sum _{k=1}^K |\delta _{jk}|, \end{aligned}$$

where

$$\begin{aligned} L^{ora}(\Xi ) = \prod _{i=1}^N \int \left( \prod _{j=1}^J \left( \exp ((a_j\theta + d_j + \delta _{j\xi _i})Y_{ij})/(1+\exp (a_j\theta + d_j + \delta _{j\xi _i}))\right) \right) \phi (\theta \vert \mu _{\xi _i}, \sigma ^2_{\xi _i}) d\theta \end{aligned}$$

and $\Xi$ includes the parameters in $\Delta$ except for those in $\varvec{\nu }$. The tuning parameter $\lambda$ is chosen by BIC. The FPR and TPR for detecting DIF items are also calculated under this setting and compared with those from the proposed method where $\xi _i$s are unknown.

**Classification of respondents.** We then consider the classification of respondents based on the MAP estimate. Again, we assume the number of latent focal groups *K* is known. We calculate the average classification error rate, given by the fraction of incorrect classifications to the overall number of classifications averaged over the number of replications:

$$\begin{aligned} \text {error}_{MAP} = \frac{1}{N} \sum _{i=1}^N {[}{\hat{\xi }}_{\text {MAP},i} \ne \xi _i], \end{aligned}$$

where

$$\begin{aligned} {\hat{\xi }}_{\text {MAP}, i} = \mathop {\text {arg max}}\limits _{k \in \{0, 1,..., K\}} {\hat{P}}(\hat{\xi } = k|{\textbf{y}}_i) \end{aligned}$$

and ${\hat{P}}({\hat{\xi }} = k | {\textbf{y}}_i)$ is the posterior probability of category *k* given in (6). We notice that there is a label-switching problem under the setting with $K = 2$ when calculating the classification error. This problem is solved by a post-hoc label switching based on the estimated $\nu _1$ and $\nu _2$, using the ordering information that class 2 is larger than class 1, i.e., $\nu _2 > \nu _1$. We also calculate the MAP classification error under the true model and compare it with the classification error of the proposed method.

**Parameter estimation accuracy.** We further evaluate the accuracy of our final estimator ${\hat{\Delta }}^{({{\hat{\lambda }}})}$, assuming that *K* is known. For each unknown parameter, the root mean square error (RMSE) and the absolute bias are calculated based on the 100 replications.

### Results

The classification performance in the simulation study is presented in Tables 1 and 2, which display the respondent and item classification accuracy for the two-group and three-group settings, respectively. First, it is observed that the classification error is predominantly influenced by the number of items, with larger item sizes resulting in better respondent classification performance. This observation is consistent with previous literature on DIF detection using IRT models (Chen et al., 2022; Kuha and Moustaki, 2015).

For respondent classification in the two-group setting, we observe in Table 1 that the classification error is small for all simulation scenarios, and always better than a naïve classifier that assigns all respondents to the reference group. This is true even when the focal group only consists of 10% of the respondents. As the proportion in the focal group (in Table 1 denoted by $\pi$) increases, the proposed method’s enhancement over the naïve classifier, which assigns all respondents to the baseline group, becomes more pronounced. We also note that the AUC values increase when the proportion of respondents in the focal group increases, but the increments are small. Table 1 furthermore shows that the classification accuracy is only slightly worse when using the estimated parameters compared to when the true model parameters are used.

For item classification in the two-group setting, the true positive rates are very high, and with no item being falsely flagged as a DIF item, across all scenarios. This suggests that the proposed framework is effective in identifying DIF items and minimizing false positives for various numbers of items and proportions in the focal group. We also note that the oracle estimator performance is only slightly better, i.e., knowing the group membership of the respondents only leads to marginal improvement in item classification accuracy.

In the three-group setting, Table 2 shows that the classification error rates are generally higher than those observed in the two-group case, which is expected given the increased complexity of the DIF detection problem when more than two groups are involved. However, note that the classification error is always clearly smaller than the naïve classifier that assigns all of the respondents to the reference group. This increase in classification performance is particularly clear in the simulation scenarios with $J=50$. We also observe that the AUC values for classes 2 and 3 are within reasonable ranges, suggesting that the proposed method is capable of adequately classifying respondents even in more challenging settings. The TPR and FPR values for item classification in the three-group setting are furthermore high for both class 2 and class 3 items and with no misclassified DIF-free item. This further supports the effectiveness of the proposed framework in identifying DIF items across different group configurations.

**Table 1:** Respondent and item classification accuracy under different simulation scenarios for the two-group case.

				$$N=1,000$$		
		$J=25$			$J=50$	
Evaluation measure	$\pi =0.1$	$\pi =0.2$	$\pi =0.5$.	$\pi =0.1$	$\pi =0.2$	$\pi =0.5$
Classification error	0.078	0.141	0.256	0.071	0.113	0.179
Classification error true	0.077	0.140	0.252	0.069	0.111	0.175
AUC	0.796	0.817	0.824	0.896	0.903	0.904
AUC true	0.813	0.827	0.830	0.903	0.907	0.908
TPR	0.957	0.948	0.948	0.997	0.993	0.996
FPR	0	0	0	0	0	0
TPR oracle	1	1	1	1	1	1
FPR oracle	0	0	0	0	0	0

				$$N=5,000$$		
		$J=25$			$J=50$	
Evaluation measure	$\pi =0.1$	$\pi =0.2$	$\pi =0.5$	$\pi =0.1$	$\pi =0.2$	$\pi =0.5$
Classification error	0.087	0.161	0.259	0.074	0.124	0.177
Classification error true	0.086	0.160	0.253	0.073	0.122	0.174
AUC	0.809	0.812	0.821	0.897	0.901	0.904
AUC true	0.819	0.819	0.827	0.902	0.904	0.907
TPR	0.953	0.947	0.950	0.995	0.993	0.996
FPR	0	0	0	0	0	0
TPR oracle	1	1	1	1	1	1
FPR oracle	0	0	0	0	0	0

The classification error and AUCs present respondent classification performance, where ‘AUC true’ gives the results using the true parameter values. The TPRs and FPRs present the results for item classification, where ‘TPR oracle’ and ‘FPR oracle’ are the performance of the oracle estimator.

**Table 2:** Respondent and item classification accuracy under different simulation scenarios for the three-group case.

	$N=1,000$		$N=5,000$	
Evaluation measure	$J=25$	$J=50$	$J=25$	$J=50$
Classification error	0.391	0.353	0.397	0.357
Classification error true	0.393	0.355	0.400	0.357
AUC class 2	0.725	0.777	0.723	0.764
AUC class 3	0.782	0.812	0.775	0.822
AUC class 2 true	0.743	0.794	0.742	0.779
AUC class 3 true	0.807	0.836	0.794	0.838
TPR class 2	0.925	0.971	0.931	0.960
TPR class 3	1	1	1	1
FPR class 2	0	0	0	0
FPR class 3	0	0	0	0
TPR class 2 oracle	1	0.95	1	1
TPR class 3 oracle	1	1	1	1
FPR class 2 oracle	0	0	0	0
FPR class 3 oracle	0	0	0	0

The classification error and AUCs present respondent classification performance, where ‘AUC true’ gives the results using the true parameter values. The TPRs and FPRs present the results for item classification, where ‘TPR oracle’ and ‘FPR oracle’ are the performance of the oracle estimator.

**Fig. 2:**
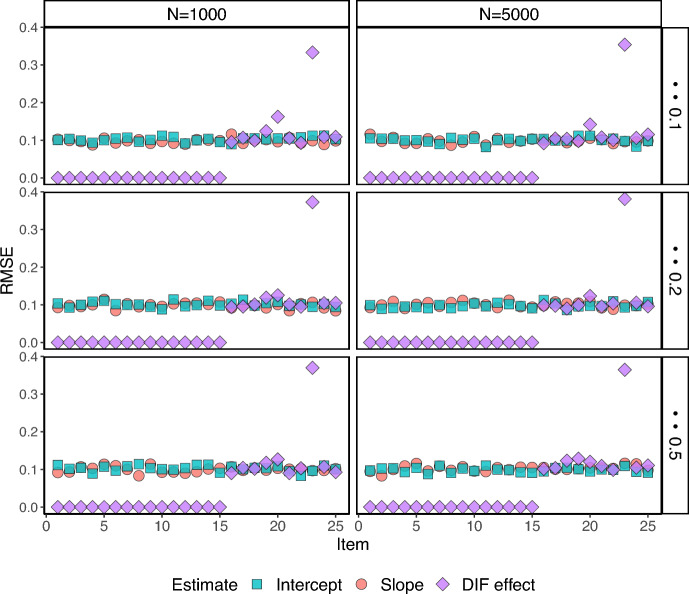
RMSE for $J=25$ under the 2-group setting.

**Fig. 3:**
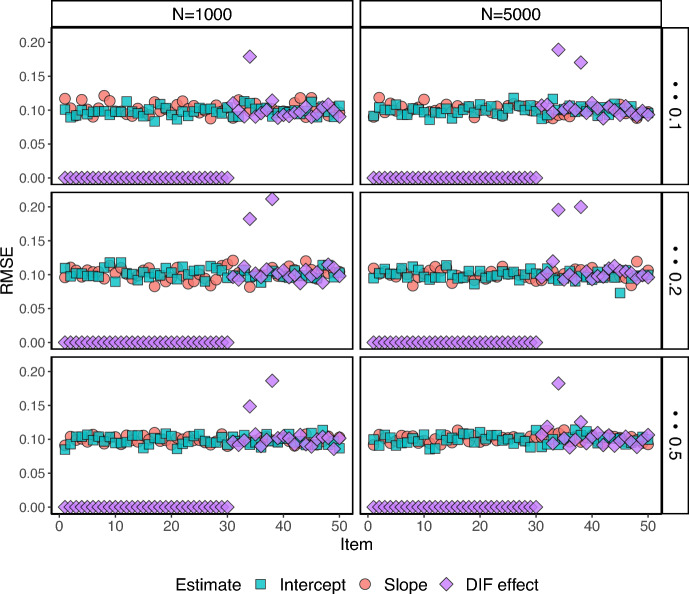
RMSE for $J=50$ under the 2-group setting.

Figures 2 and 3 show the RMSEs (Root Mean Squared Errors) for all the item parameter estimates for the two-group setting ($K=1$). The RMSEs are small for all estimates across the items, with the exemption of one or two items that show larger RMSEs for the estimated DIF parameter. The RMSEs for the estimated DIF parameters for the DIF-free items are zero as the proposed estimation procedure successfully classifies the DIF items. We also see that the number of items and proportion of respondents in the focal group have essentially no influence on the RMSE, for the configurations considered in this simulation study.

**Fig. 4:**
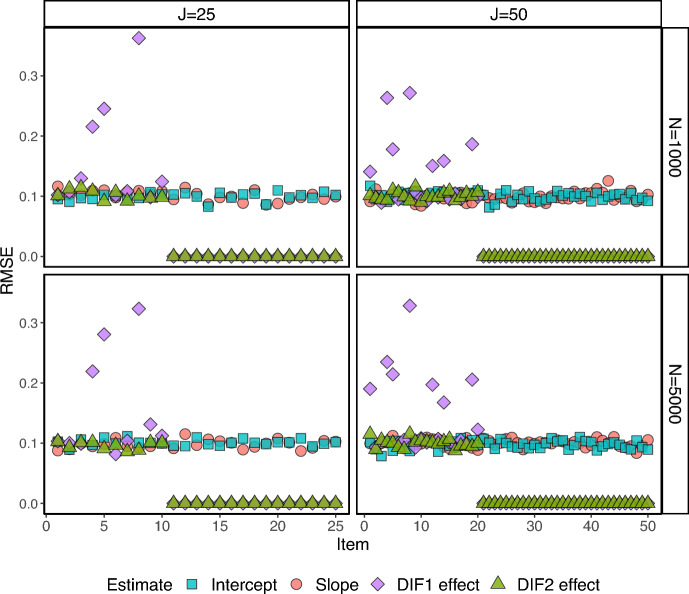
The RMSEs for under the 3-group setting.

In Fig. 4, we can observe the RMSEs specifically for the three-group scenario. It is important to note that in this case, there exist two DIF effects, one for each focal group. For the first focal group, the true DIF effects are drawn from values in the range [0.5 – 1] and for the second focal group, they are drawn from the range [1 – 1.5]. The increased difficulty of estimating smaller DIF effects is reflected by larger RMSEs for the first focal group.

In Table 3 and Table 4, the absolute bias and RMSE under the 2-group setting, averaged over the number of items of the same type, are displayed for both sample sizes and every focal group proportion $\pi$ considered. The absolute bias and RMSE are small for all parameters, and differences are very small between different values of $\pi$ and different values of *N*. The most notable difference is seen for the estimate of the focal group proportion $\pi$, where the bias and RMSE clearly decrease when the sample size increases. We also see that the DIF effect parameter is estimated with only a small bias and RMSE. In Table 5, the absolute bias and RMSE are shown for the 3-group setting. As in the two-group setting, the bias and RMSE values are small under all settings.

In summary, the simulation results presented in Tables 1–5, and Figs. 2–4 demonstrate the potential of the proposed framework for DIF analysis with unknown anchor items and comparison groups. The framework performs well in terms of respondent and item classification accuracy across a range of scenarios, and with good parameter recovery, suggesting its applicability in various real-world settings.

## Real Data Analysis

To illustrate the proposed method, we analyse a mathematics test from a midwestern university in the United States. This data set has been analysed in both Bolt et al. (2002) and De Boeck et al. (2011). The data contains 3000 examinees answering 26 binary-scored items. The original dataset contains two test forms, with 8 items in common.^1^ Six of the common items are of particular interest as they are positioned at the end of the test. Bolt et al. (2002) hypothesised the existence of two latent classes: one speeded class that answered end-of-test items with insufficient time, and another non-speeded class. The identification of speeded items was conducted using a two-form design. Specifically, they examined common items across two test administrations, where the common items were placed at the end of the test in one form and earlier in the other form. By estimating the item difficulty for the end-of-test common items and comparing it to the difficulty estimates from the other form, they were able to quantify the DIF effect. Our goal is to detect the DIF items, i.e., the speeded items, and classify respondents into latent classes. Thanks to our procedure, we can analyse only one form, without using information from the second form. A similar analysis using simulated data was also conducted in Robitzsch (2022) but using a Rasch mixture model.

We start by fitting the proposed model to the data for different values of *K*, which determines the number of latent classes. The BIC for a model with $K=0$, i.e. no latent classes other than the reference group, equals 117,552.2, the BIC for $K=1$ equals 92,300.1, and for $K=2$, the BIC equals 92,522.8. We therefore proceed with a model using $K=1$, i.e., two latent classes. This aligns with the two-group model considered in Bolt et al. (2002). It took the proposed EM algorithm 37.04, 144.760, and 277.390 s to converge^2^ for the 1-. 2-, and 3-class solutions, respectively.^3^ The proposed model classifies 25.8% of the respondents into the second latent class. If we interpret the two classes as a speeded and non-speeded class, this means that about 26% of the respondents belong to the speeded class. The estimated mean ability in the speeded class equals $-$0.351 with the estimated standard deviation equal to 1.075. Since the reference group (the non-speeded class) has a prespecified ability mean and standard deviation equal to 0 and 1, respectively, our results therefore indicate that the speeded class has a lower ability on average compared to the non-speeded class. These findings align closely with the results presented in Bolt et al. (2002).

**Table 3:** Average absolute bias and RMSE over all items by estimated parameter type, when $J=25$, $N=1000$, and $5000$, under the 2-group setting.

		$N=1,000$		$N=5,000$	
Scenario	Parameter	Abs. bias	RMSE	Abs. bias	RMSE
$\pi =0.1$	$d$	0.078	0.098	0.079	0.099
	$a$	0.082	0.103	0.081	0.100
	$\delta$	0.040	0.053	0.041	0.053
	$\mu$	0.087	0.114	0.077	0.098
	$\sigma$	0.074	0.093	0.088	0.108
	$\pi$	0.035	0.040	0.017	0.022
$\pi =0.2$	$d$	0.078	0.098	0.081	0.101
	$a$	0.081	0.101	0.078	0.098
	$\delta$	0.041	0.053	0.041	0.052
	$\mu$	0.081	0.105	0.082	0.107
	$\sigma$	0.082	0.101	0.086	0.106
	$\pi$	0.050	0.056	0.022	0.027
$\pi =0.5$	$d$	0.079	0.100	0.081	0.102
	$a$	0.082	0.103	0.079	0.099
	$\delta$	0.040	0.052	0.042	0.055
	$\mu$	0.084	0.104	0.076	0.093
	$\sigma$	0.075	0.094	0.080	0.101
	$\pi$	0.040	0.049	0.030	0.037

**Table 4:** Average absolute bias and RMSE over all items by estimated parameter type, when $J=50$, $N=1000$, and $5000$ under the 2-group setting.

		$N=1,000$		$N=5,000$	
Scenario	Parameter	Abs. bias	RMSE	Abs. bias	RMSE
$\pi =0.1$	$d$	0.082	0.102	0.080	0.100
	$a$	0.078	0.098	0.079	0.100
	$\delta$	0.032	0.041	0.033	0.043
	$\mu$	0.081	0.102	0.079	0.096
	$\sigma$	0.078	0.099	0.077	0.096
	$\pi$	0.026	0.030	0.013	0.016
$\pi =0.2$	$d$	0.081	0.102	0.080	0.100
	$a$	0.081	0.102	0.079	0.099
	$\delta$	0.034	0.044	0.034	0.044
	$\mu$	0.081	0.100	0.077	0.101
	$\sigma$	0.082	0.097	0.084	0.106
	$\pi$	0.034	0.038	0.014	0.018
$\pi =0.5$	$d$	0.079	0.099	0.081	0.101
	$a$	0.079	0.098	0.080	0.100
	$\delta$	0.032	0.042	0.032	0.042
	$\mu$	0.091	0.112	0.078	0.097
	$\sigma$	0.073	0.093	0.081	0.106
	$\pi$	0.030	0.038	0.021	0.025

**Table 5:** Average absolute bias and RMSE over all items by estimated parameter type, $N=1000$ and $5000$, under the 3-group setting.

		$N=1,000$		$N=5,000$	
Scenario	Parameter	Abs. bias	RMSE	Abs. bias	RMSE
$J=25$	$d$	0.081	0.101	0.078	0.098
	$a$	0.080	0.100	0.080	0.101
	$\delta _2$	0.046	0.064	0.045	0.062
	$\delta _3$	0.033	0.041	0.031	0.038
	$\mu _2$	0.084	0.103	0.079	0.095
	$\mu _3$	0.074	0.094	0.073	0.094
	$\sigma _2$	0.085	0.107	0.083	0.103
	$\sigma _3$	0.081	0.099	0.084	0.103
	$\pi _2$	0.060	0.075	0.045	0.057
	$\pi _3$	0.045	0.057	0.044	0.054
$J=50$	$d$	0.080	0.099	0.079	0.100
	$a$	0.08	0.101	0.078	0.098
	$\delta _2$	0.038	0.052	0.039	0.057
	$\delta _3$	0.033	0.040	0.032	0.040
	$\mu _2$	0.083	0.100	0.083	0.104
	$\mu _3$	0.072	0.090	0.080	0.097
	$\sigma _2$	0.076	0.092	0.085	0.107
	$\sigma _3$	0.081	0.103	0.072	0.096
	$\pi _2$	0.064	0.079	0.055	0.067
	$\pi _3$	0.044	0.056	0.035	0.046

In Table 6 we give the estimated item parameters from the educational test data. The estimated item discrimination and easiness parameters, ${\hat{a}}$ and ${\hat{d}}$ respectively, are provided together with the estimated DIF effect ${\hat{\delta }}$. Common items are denoted by asterisks. For the majority of the items, the DIF effects are estimated to be zero, indicating that these items do not exhibit any significant measurement bias between different groups. However, items 20-26 exhibit non-zero DIF effects, suggesting that these items might be functioning differently for the two latent classes. Among these, items 20, 21, 22, 23, and 24 are also common items, which may require further investigation to ensure fair assessment across test administrations. Since the DIF effect for these end-of-test items is all negative, it suggests that these items become more difficult for the second latent class. This class could therefore consist of respondents that ran out of time and had insufficient time to answer these items. This is known as a speededness effect. As a result, the item difficulty is inflated, which could lead to biased subsequent analyses. The presence of non-zero DIF effects for some end-of-test items highlights the need to scrutinize these items more closely and potentially revise the test administration to minimize the impact of speededness. For instance, increasing the allocated time for the test or redistributing the items more evenly throughout the test could help alleviate the speededness effect and create a more unbiased assessment.

**Table 6:** Estimated item easiness and DIF effects for the detected DIF items.

Item	${\hat{a}}$	${\hat{d}}$	${\hat{\delta }}$	Item	${\hat{a}}$	${\hat{d}}$	${\hat{\delta }}$
1	1.298	2.987	0	14	0.746	0.941	0
2	1.287	1.584	0	15	0.471	$-$1.646	0
3	0.552	2.968	0	16*	1.507	$-$0.526	0
4	0.878	0.707	0	17	1.271	1.843	0
5	1.336	2.247	0	18	1.249	$-$2.588	0
6	0.973	0.822	0	19	1.071	0.944	0
7	0.669	0.296	0	20*	1.017	0.455	$-$1.411
8	0.687	$-$0.882	0	21*	1.425	1.324	$-$1.808
9	1.268	0.908	0	22*	0.664	$-$0.608	$-$1.104
10	0.874	2.101	0	23*	0.767	$-$0.063	$-$0.624
11*	0.984	$-$0.575	0	24*	0.929	$-$0.639	$-$0.885
12	0.912	$-$0.804	0	25	1.247	$-$2.020	$-$1.019
13	1.040	0.917	0	26*	1.340	$-$0.079	$-$0.851

The asterisks denote the common items.

## Concluding Remarks

In this paper, we presented a comprehensive framework for DIF analysis that overcomes several limitations of existing methods. Our approach can deal with the situation in which both anchor items and comparison groups are unknown, a setting commonly encountered in real-world applications. The use of latent classes in our approach allows us to model heterogeneity among the observations. In this sense, our approach relates to an exploratory dimensionality analysis where there is, in addition to the primary latent dimension, a second dimension that is treated as unknown. In our empirical analysis, this additional dimension is labeled as a speededness effect. In addition to modeling the additional latent dimension(s), the proposed regularised estimator enables us to identify DIF items and quantify their effect on the intercept parameter of the model. We also propose an efficient EM algorithm for the estimation of the model parameters.^4^ One merit of our framework is its flexibility. While focusing on the 2-PL model as the baseline model, our approach can be easily extended to accommodate other widely used IRT models, such as the Rasch model and the proportional odds model. We can also allow the baseline model to be a multidimensional IRT model, as shown in Sect. 1.6. Our framework is furthermore able to accommodate more than two comparison groups, allowing DIF effects to vary between the groups. Lastly, the proposed method can be extended as shown in Section 2.6 to detect non-uniform DIF. This flexibility makes our framework applicable to a wide range of contexts.

Although our approach shows promising results, there are still several limitations to be addressed in future research. For example, we do not provide confidence intervals for the DIF effect parameters which would be useful for practitioners and researchers in interpreting the magnitude and significance of the DIF effects. In Chen et al. (2023) for example, where the comparison groups are known but the anchor items are unknown, the distribution of ${\hat{\delta }}_{jk} - \delta _{jk}$ is approximated by Monte Carlo simulation to yield valid statistical inference. This procedure does in essence apply to our case as well. In addition, we have not linked the latent classes to covariates, as in for example Vermunt (2010) and Vermunt and Magidson (2021). By doing so, researchers can gain insights into the underlying characteristics of the different classes and better understand the factors that may contribute to DIF. This would enhance the interpretability of the results and help identify potential sources of DIF that could be addressed in the development of assessment instruments. To address this limitation, future research could explore the integration of covariates within a structural equation modeling (SEM) framework. This would enable the simultaneous modelling of both the measurement model (i.e., the IRT model) and the structural model (i.e., relationships between latent variables and covariates). Incorporating covariates in this manner would not only improve the interpretability of the results but could also provide a more comprehensive understanding of the relationships between the items, latent traits, and potential sources of DIF. Our framework could also be extended to accommodate non-uniform DIF, i.e., DIF in the slope parameter, such as in Wang et al. (2023) that considers a multidimensional IRT model with known comparison groups and unknown anchor items.

In this study, we focus on the Lasso penalty for its simplicity, computational efficiency, and well-documented ability to perform both variable selection and regularisation. The Lasso’s convex optimisation problem is easier to solve computationally than some non-convex penalties like the SCAD (Fan and Li, 2001) and the Minimax Concave Penalty (MCP; Zhang, 2010). We acknowledge that the Lasso penalty can introduce some bias into parameter estimates. However, in our proposed method we use the Lasso for model selection. As we thereafter refit the selected model there will be no bias, asymptotically, supposing that the model selection based on the Lasso is consistent (Zhao et al., 2021). Alternative penalties, including the adaptive Lasso (Zou, 2006), SCAD, and MCP, have their own merit. However, they also come with some challenges, especially in terms of computational complexity and algorithm stability. We, therefore, argue that the Lasso penalty is a suitable choice for the proposed model and its identifying assumptions. We believe it would be interesting in the future to compare the performance of estimators with different penalty functions under the current latent DIF setting.

Our proposed framework provides a powerful tool for DIF analysis with unknown anchor items and comparison groups. The framework has the potential to inform the development of fair and unbiased assessments. Future research can build upon our approach by addressing the limitations and exploring other applications. In terms of the potential impact of our work, the framework could be particularly beneficial in specific contexts, such as educational assessment, where identifying and addressing DIF is critical to ensure that tests fairly measure students’ abilities across heterogeneous populations, thereby promoting equal access to educational opportunities. It could also be considered in employment selection, where unbiased assessment instruments are crucial to creating a diverse and inclusive workforce that complies with legal requirements related to fairness in employment practices (Ployhart and Holtz, 2008; Hough et al., 2001). Another application is psychological evaluations, where accurate identification of DIF can help improve diagnostic tools and treatment recommendations, leading to better outcomes for individuals from diverse backgrounds (Teresi et al., 2021). By addressing the limitations and further refining our approach, this framework has the potential to contribute to the development of more fair assessment practices in these and other domains, ultimately benefiting a wide range of stakeholders.

### Supplementary Information

Below is the link to the electronic supplementary material.

Supplementary file 1 (pdf 68 KB)

## Figures and Tables

**Algorithm 3:**
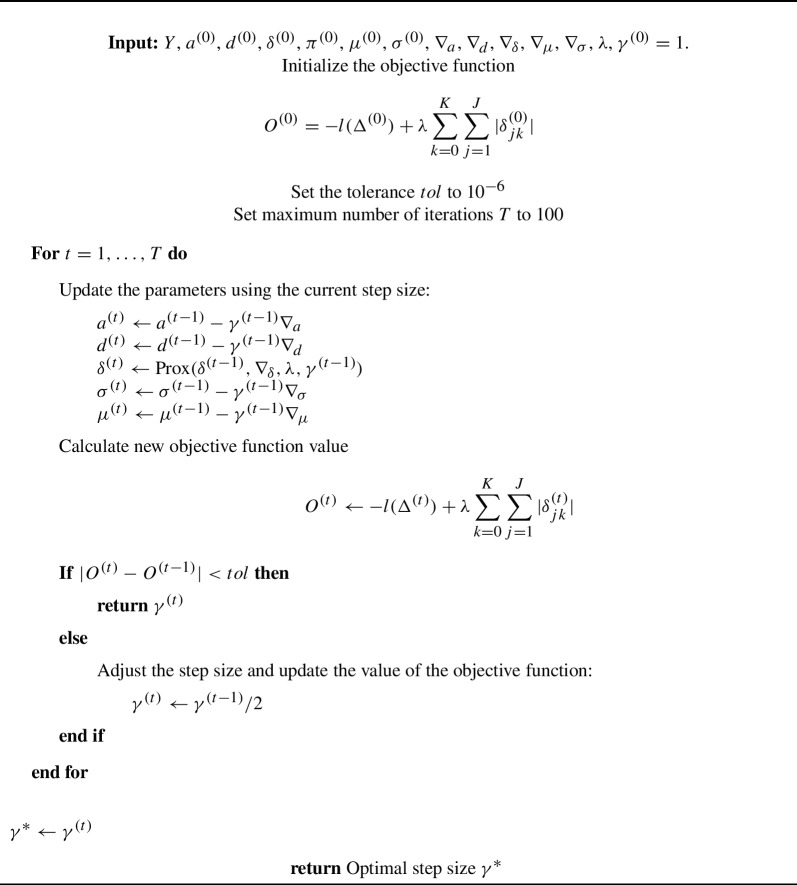
Line Search Algorithm

**Algorithm 4:**
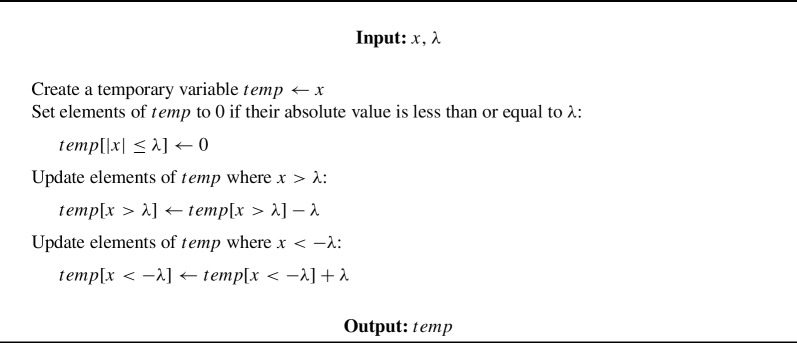
Soft Threshold Function

**Algorithm 5:**
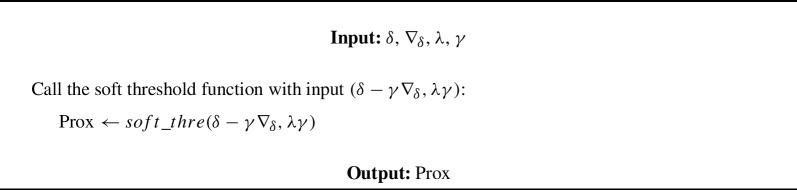
Proximal Gradient Function

## Gradients for the Proximal Gradient Descent

In the M-step of the proposed EM-algorithm, we implement a proximal gradient descent. This algorithm requires the gradients of the objective function, which for the proposed model can be expressed as

17 $$\begin{aligned} \frac{\partial l(\Delta )}{\partial \eta _j} = \sum _{i=1}^N \int \frac{\partial \varphi _{ij}}{\partial \eta _j} \bigg [ y_{ij} - P(Y_{ij} = 1 | \theta _i, \xi _i) \bigg ] \phi (\theta _i; \mu _k, \sigma ^2_k) d \theta _i, \end{aligned}$$

where $\eta _j$ is a generic notation for the item parameters $(a_j, d_j, \delta _{jk})$, and $\varphi _{ij} = d_j + a_j\theta _i + \delta _{j\xi _i}$,

To give an example, we give the explicit expressions for the two-group case. We start by parametrising the latent construct of the focal group as $\theta _2 = \mu _2 + \sigma _2 \theta _1$, where $\mu$ and $\sigma$ is the mean and standard deviation of the latent construct in the focal group, and $\theta _1 \sim {\mathcal {N}}(0,1)$ is the latent construct in the reference group. We have that $\varphi _{ij}^{(1)} = d_j + a_j \theta _{i,1}$ for the reference group and $\varphi _{ij}^{(2)} = d_j + \delta _j + a_j \theta _{i,2}$ for the focal group. The partial derivatives in (17) are given by

18 $$\begin{aligned}{} & {} \frac{\partial \varphi _{ij}^{(1)}}{\partial d_j} = 1 \quad \frac{\partial \varphi _{ij}^{(2)}}{\partial d_j} = 1 \frac{\partial \varphi _{ij}^{(1)}}{\partial a_j} = \theta _1 \quad \frac{\partial \varphi _{ij}^{(2)}}{\partial a_j} = \mu _2 + \sigma _2 \theta _{i,1} \nonumber \\{} & {} \frac{\partial \varphi _{ij}^{(1)}}{\partial \delta _{j2}} = 0 \quad \frac{\partial \varphi _{ij}^{(2)}}{\partial \delta _{j2}} = 1 \frac{\partial \varphi _{ij}^{(1)}}{\partial \mu _2} = 0 \quad \frac{\partial \varphi _{ij}^{(2)}}{\partial \mu _2} = 1 \frac{\partial \varphi _{ij}^{(1)}}{\partial \sigma _2} = 0 \quad \frac{\partial \varphi _{ij}^{(2)}}{\partial \sigma _2} = \theta _1 \end{aligned}$$

## The Line Search Procedure

The implemented line search algorithm attempts to find an appropriate step size for the proximal gradient descent implemented in the M-step of the proposed EM algorithm. It does so by iteratively adjusting the step size until the change in the objective function (i.e., the penalised log-likelihood) is within a specified tolerance. The algorithm starts with an initial step size and iteratively reduces it by a factor (in this case, dividing by 2) until the new objective function value satisfies the tolerance condition. If the maximum number of iterations is reached without finding a satisfactory step size, the algorithm returns the current step size. The implemented line search algorithm is given in the following Line Search Algorithm.

## The Soft-Thresholding Procedure

The soft threshold function and the proximal gradient function are used to identify the DIF-free items, i.e., the anchor items, from the data. The soft threshold function takes a vector *x* and a scalar $\lambda$ as inputs and applies element-wise thresholding to *x*. It sets elements with absolute values less than or equal to $\lambda$ to zero, subtracts $\lambda$ from elements greater than $\lambda$, and adds $\lambda$ to elements less than $-\lambda$. The proximal gradient function takes the (estimated) DIF effect $\delta$, its gradient $\nabla _x$, a regularisation parameter $\lambda$, and a step size $\gamma$ as inputs. It calls the soft threshold function with the updated vector $\delta - \gamma \nabla _\delta$ and the product $\lambda \gamma$. The output of the proximal gradient function is the updated DIF effect parameter estimate after applying the soft threshold function. These algorithms are summarised below.

## The Closed-Form Solution of the Latent Class Proportion

Using the Lagrange multiplier method, we obtain

$$\begin{aligned} \nu _k^{(t+1)} = \frac{\sum _{i=1}^n \gamma _{ik}^{(t)}}{n}, \end{aligned}$$

where $$\gamma _{ik}^{(t)} = P(\xi _i = k | {\textbf{y}}_i)$$

We can see this from the following argument. Given that

$$\begin{aligned} D_t(\Delta _1) = -\sum _{i=1}^N {\mathbb {E}}\left[ \log \left( \nu _{\xi _i} \big \vert {\textbf{Y}}_i, \Delta ^{(t)}\right) \right] . \end{aligned}$$

The expectation can be expressed as

$$\begin{aligned} {\mathbb {E}}\left[ \log (\nu _{\xi _i}) \big \vert {\textbf{Y}}_i, \Delta ^{(t)}\right] = \sum _{k=0}^K P(\xi _i = k \big \vert {\textbf{Y}}_i, \Delta ^{(t)}) \log (\nu _k). \end{aligned}$$

Thus, we have

$$\begin{aligned} D_t(\Delta _1) = -\sum _{i=1}^N \sum _{k=0}^K \gamma _{ik}^{(t)} \log (\nu _k), \end{aligned}$$

where $$\gamma _{ik}^{(t)} = P(\xi _i = k \big \vert {\textbf{Y}}_i, \Delta ^{(t)})$$.

Construct the Lagrangian function with the constraints:

$$\begin{aligned} {\mathcal {L}}(\Delta _1, \varvec{\alpha }, \beta ) = D_t(\Delta _1) + \sum _{k=0}^K \alpha _k (\nu _k - \epsilon _k) + \beta \left( \sum _{k=0}^K \nu _k - 1\right) , \end{aligned}$$

where $$\alpha _k \ge 0$$ for $$k = 0, 1, \dots , K$$.

Now, take the partial derivative of the Lagrangian function with respect to $\nu _k$:

$$\begin{aligned} \frac{\partial {\mathcal {L}}}{\partial \nu _k} = -\sum _{i=1}^n \gamma _{ik}^{(t)}\frac{1}{\nu _k} + \alpha _k + \beta , \quad k = 0, 1, \dots , K. \end{aligned}$$

Setting the partial derivatives to zero, we get

$$\begin{aligned} -\sum _{i=1}^n \gamma _{ik}^{(t)}\frac{1}{\nu _k} + \alpha _k + \beta = 0, \quad k = 0, 1, \dots , K. \end{aligned}$$

From the above equation, we have:

$$\begin{aligned} \nu _k^{(t+1)} = \frac{\sum _{i=1}^n \gamma _{ik}^{(t)}}{-\alpha _k - \beta }, \quad k = 0, 1, \dots , K. \end{aligned}$$

To find the values of $\alpha _k$ and $\beta$, we need to apply the constraint $\sum _{k=0}^K \nu _k = 1$. Plugging in the expression for $\nu _k^{(t+1)}$, we have

$$\begin{aligned} \sum _{k=0}^K \frac{\sum _{i=1}^n \gamma _{ik}^{(t)}}{-\alpha _k - \beta } = 1. \end{aligned}$$

Since the constraint only involves the sum of the $\nu _k^{(t+1)}$, we can eliminate the Lagrange multipliers $\alpha _k$ by normalizing the solution:

$$\begin{aligned} \nu _k^{(t+1)} = \frac{\sum _{i=1}^n \gamma _{ik}^{(t)}}{\sum _{k=0}^K \sum _{i=1}^n \gamma _{ik}^{(t)}}, \quad k = 0, 1, \dots , K. \end{aligned}$$

However, since $\sum _{k=0}^K \gamma _{ik}^{(t)} = 1$ for all $i = 1, 2, \dots , n$, the denominator simplifies to the total number of observations *N*. Therefore, we have the closed-form solution:

$$\begin{aligned} \nu _k^{(t+1)} = \frac{\sum _{i=1}^n \gamma _{ik}^{(t)}}{n}, \quad k = 0, 1, \dots , K. \end{aligned}$$

## Footnotes

1 The original dataset contains 36 items, with 12 common items. We follow the procedure in Bolt et al. (2002) and only analyse items 1-18 and 29-36. In this analysis, items 19-26 therefore actually occupied item position 29-36.

2 In our implementation of the proposed EM algorithm, we stop the iterations when the increase in the log-likelihood is smaller than $10^{-4}$.

3 CPU configuration: 11th Gen Intel(R) Core(TM) i7-1165G7 2.80GHz 3200MHz.

4 The R code for the proposed method is available from https://github.com/gabrieltwallin/LatentDIF/
