# Supplementary material for: DIF Analysis with Unknown Groups and Anchor Items
Source: Psychometrika. 2024 Feb 21;89(1):267–95. doi: 10.1007/s11336-024-09948-7 (PMC11062998; doi:10.1007/s11336-024-09948-7)
Supplement: Supplementary file 1 — (pdf 68 KB) [file 11336_2024_9948_MOESM1_ESM.pdf]

Supplementary Material

1 The True Parameter Values in the Simulation Study

In Tables 1–4, the true item parameter values for the simulation study are displayed.

Table 1: The true item parameter values for simulation scenarios where $J = 25$ and $K = 1$.

Item	a_j	d_j	δ_j
Item 1	1.177	-0.276	0
Item 2	1.108	0.763	0
Item 3	0.509	-1.682	0
Item 4	1.291	-0.173	0
Item 5	1.291	-0.217	0
Item 6	0.559	-0.724	0
Item 7	1.306	1.382	0
Item 8	1.433	-0.990	0
Item 9	1.245	-1.536	0
Item 10	0.867	-1.761	0
Item 11	0.950	-0.236	0
Item 12	1.104	0.120	0
Item 13	0.729	-0.923	0
Item 14	0.973	-0.567	0
Item 15	1.202	-1.889	0
Item 16	0.890	-1.914	1.163
Item 17	0.504	0.781	1.114
Item 18	1.163	1.767	1.090
Item 19	1.282	-1.924	0.746
Item 20	0.756	-0.841	0.741
Item 21	0.947	-1.548	1.226
Item 22	0.604	-0.049	1.046
Item 23	1.008	1.345	0.519
Item 24	0.544	-1.118	1.034
Item 25	1.452	-0.851	0.992

Table 2: The true item parameter values for simulation scenarios where $J = 50$ and $K = 1$.

Item	a_j	d_j	δ_j
Item 1	1.177	0.652	0
Item 2	1.108	0.454	0
Item 3	0.509	0.360	0
Item 4	1.291	-1.016	0
Item 5	1.291	-1.035	0
Item 6	0.559	0.905	0
Item 7	1.306	0.183	0
Item 8	1.433	-1.926	0
Item 9	1.245	0.137	0
Item 10	0.867	-0.032	0
Item 11	0.950	-0.038	0
Item 12	1.104	1.435	0
Item 13	0.729	-0.887	0
Item 14	0.973	0.456	0
Item 15	1.202	-1.121	0
Item 16	0.890	-1.947	0
Item 17	0.504	-1.601	0
Item 18	1.163	0.772	0
Item 19	1.282	0.956	0
Item 20	0.756	-0.193	0
Item 21	0.947	-0.442	0
Item 22	0.604	0.428	0
Item 23	1.008	1.355	0
Item 24	0.544	1.386	0
Item 25	1.452	-1.968	0
Item 26	0.931	-1.265	0
Item 27	1.191	-1.173	0
Item 28	0.580	0.776	0
Item 29	0.957	1.251	0
Item 30	0.946	-0.031	0
Item 31	0.819	-0.554	0.796
Item 32	1.345	0.823	1.046
Item 33	0.752	-0.580	1.473
Item 34	0.616	-1.402	0.664
Item 35	0.560	-1.980	0.815
Item 36	0.941	-0.283	1.346
Item 37	1.030	-1.211	1.169
Item 38	0.769	-1.741	0.675
Item 39	0.858	-1.802	1.406
Item 40	0.528	0.074	1.059
Item 41	0.522	1.417	1.397
Item 42	1.195	-1.600	0.827
Item 43	1.442	0.115	1.258
Item 44	0.519	0.367	0.869
Item 45	0.790	-0.784	1.047
Item 46	0.613	-1.283	1.271
Item 47	0.988	1.574	1.268
Item 48	1.336	0.549	1.414
Item 49	0.720	1.420	0.833
Item 50	0.787	1.832	1.040

Table 3: The updated item parameter values for simulation scenarios where $J = 25$ and $K = 2$.

Item	a_j	d_j	δ_{j2}	δ_{j3}
Item 1	1.177	-0.276	0.831	1.245
Item 2	1.108	0.763	0.807	1.429
Item 3	0.509	-1.682	0.795	1.139
Item 4	1.291	-0.173	0.623	1.307
Item 5	1.291	-0.217	0.621	1.110
Item 6	0.559	-0.724	0.863	1.007
Item 7	1.306	1.382	0.773	1.050
Item 8	1.433	-0.990	0.509	1.347
Item 9	1.245	-1.536	0.767	1.369
Item 10	0.867	-1.761	0.746	1.226
Item 11	0.950	-0.236	0	0
Item 12	1.104	0.120	0	0
Item 13	0.729	-0.923	0	0
Item 14	0.973	-0.567	0	0
Item 15	1.202	-1.889	0	0
Item 16	0.890	-1.914	0	0
Item 17	0.504	0.781	0	0
Item 18	1.163	1.767	0	0
Item 19	1.282	-1.924	0	0
Item 20	0.756	-0.841	0	0
Item 21	0.947	-1.548	0	0
Item 22	0.604	-0.049	0	0
Item 23	1.008	1.345	0	0
Item 24	0.544	-1.118	0	0
Item 25	1.452	-0.851	0	0

Table 4: The true item parameter values for simulation scenarios where $J = 50$ and $K = 2$.

Item	a_j	d_j	δ_{j2}	δ_{j3}
Item 1	1.177	0.652	0.648	1.116
Item 2	1.108	0.454	0.773	1.008
Item 3	0.509	0.360	0.987	1.116
Item 4	1.291	-1.016	0.582	1.053
Item 5	1.291	-1.035	0.658	1.380
Item 6	0.559	0.905	0.923	1.098
Item 7	1.306	0.183	0.834	1.277
Item 8	1.433	-1.926	0.587	1.210
Item 9	1.245	0.137	0.953	1.150
Item 10	0.867	-0.032	0.779	1.105
Item 11	0.950	-0.038	0.948	1.451
Item 12	1.104	1.435	0.663	1.095
Item 13	0.729	-0.887	0.879	1.410
Item 14	0.973	0.456	0.684	1.446
Item 15	1.202	-1.121	0.773	1.022
Item 16	0.890	-1.947	0.885	1.366
Item 17	0.504	-1.601	0.884	1.197
Item 18	1.163	0.772	0.957	1.316
Item 19	1.282	0.956	0.666	1.124
Item 20	0.756	-0.193	0.770	1.292
Item 21	0.947	-0.442	0	0
Item 22	0.604	0.428	0	0
Item 23	1.008	1.355	0	0
Item 24	0.544	1.386	0	0
Item 25	1.452	-1.968	0	0
Item 26	0.931	-1.265	0	0
Item 27	1.191	-1.173	0	0
Item 28	0.580	0.776	0	0
Item 29	0.957	1.251	0	0
Item 30	0.946	-0.031	0	0
Item 31	0.819	-0.554	0	0
Item 32	1.345	0.823	0	0
Item 33	0.752	-0.580	0	0
Item 34	0.616	-1.402	0	0
Item 35	0.560	-1.980	0	0
Item 36	0.941	-0.283	0	0
Item 37	1.030	-1.211	0	0
Item 38	0.769	-1.741	0	0
Item 39	0.858	-1.802	0	0
Item 40	0.528	0.074	0	0
Item 41	0.522	1.417	0	0
Item 42	1.195	-1.600	0	0
Item 43	1.442	0.115	0	0
Item 44	0.519	0.367	0	0
Item 45	0.790	-0.784	0	0
Item 46	0.613	-1.283	0	0
Item 47	0.988	1.574	0	0
Item 48	1.336	0.549	0	0
Item 49	0.720	1.420	0	0
Item 50	0.787	1.832	0	0
